# Prescribed opioid use is associated with increased all-purpose emergency department visits and hospitalizations in community-dwelling older adults in the United States

**DOI:** 10.3389/fpsyt.2022.1092199

**Published:** 2022-12-13

**Authors:** Song Ge, Chong Tian, Liang Wu, Minhui Liu, Haidong Lu

**Affiliations:** ^1^RN-BSN Program, Department of Natural Sciences, University of Houston-Downtown, Houston, TX, United States; ^2^School of Nursing, Tongji Medical College, Huazhong University of Science and Technology, Wuhan, China; ^3^Janssen R&D, San Diego, CA, United States; ^4^Xiangya School of Nursing, Central South University, Changsha, China; ^5^Department of Epidemiology of Microbial Diseases and the Public Health Modeling Unit, Yale School of Public Health, Yale University, New Haven, CT, United States

**Keywords:** opioid, pain, emergency department, hospitalization, older adults, national survey

## Abstract

**Background:**

The geriatric and health characteristics of older adults make them more susceptible to the effects of opioids than younger groups. The number of older adults in the United States visiting the emergency department (ED) and overusing opioids has increased in recent years. Research examining their relationship is, however, limited.

**Methods:**

Using information from the 2020 National Health Interview Survey (NHIS), we included older adults aged 65 and older. To investigate the relationship between prescribed opioid use and 12-months ED visits and hospitalizations, linear regression and logistic regression models were built while adjusting for age, sex, ethnicity, education, employment, general health status, history of depression, and living arrangement.

**Results:**

Our study population consisted of 8,631 participants (mean age 74.3). Most of them were females (58.3%) and Caucasian (81.6%). About 16% of the participants used prescribed opioids over the past 12 months. Of the participants with prescribed opioid use, 65.1% of them did so to treat chronic pain. The adjusted regression models revealed that prescribed opioid use was independently and positively associated with 12-months ED visits (β = 0.22, 95% confidence interval [CI] 0.18, 0.26) and hospitalizations (Odds ratio [OR] = 3.78, 95% CI 3.29, 4.35). Other risk factors for 12-months ED visits and/or hospitalizations included advanced age, male gender, unemployment/retirement, African American ethnicity, living alone, fair or poor general health status, and history of depression.

**Discussions:**

Clinicians should screen older adults at high risk for ED visits and hospitalizations and explore multimodal pain management with them to help them reduce/stop using opioids. These efforts may decrease their chronic pain, opioid use, opioid use-related adverse health outcomes, ED visits, as well as hospitalizations.

## Introduction

The opioid crisis is a public health concern in the US (1). According to the US Centers for Disease Control and Prevention (CDC), there were 107,622 drug overdose deaths in the US in 2021, a 15% increase from that in 2020 (2). While the opioid crisis has gained national attention from multiple stakeholders, most of the attention has focused on the young group, with limited studies exclusively targeting older adults (3–5). Opioid use among older adults was understudied, although this population is more likely to receive prescribed opioids compared with other age groups (6). For those over 50, the primary source of opioids comes from doctors’ prescriptions rather than illegal drug traffickers (7). Among older adults, opioid overdose is linked to a variety of negative health outcomes, such as sedation, vertigo, falls, injuries, respiratory problems, gastrointestinal symptoms, and cognitive function impairment (8–11). In addition, the number of older adults receiving opioid overuse treatment has continually increased over the years (3, 12). As the US population continues to age (13), opioid use among older adults is expected to be an escalating public health concern affecting older adults and their families in the US.

Older adults’ demand for critical care increases with age (14). In 2018, there were an estimated 2.4 million emergency department (ED) visits and over 700,000 hospitalizations in US older adults due to falls, motor vehicle crashes, opioid overdoses, and self-harm (15). The cost associated with ED visits is higher in older adults than in other age groups (14, 16). Rather than increasing ED resources to meet the increasing demands of older adults, the emphasis should be on reducing ED visits in this population (14). While previous studies examined opioid overdoses related ED visits (17–19), to our knowledge, no study has examined all-purpose ED visits and hospitalizations related to prescribed opioid use exclusively in older adults.

Thus, the purpose of this study is to (1) assess the prevalence of prescribed opioid use in US community-dwelling older adults, (2) assess the prevalence of using prescribed opioids for treating chronic pain in US community-dwelling older adults, and (3) examine the independent relationship between prescribed opioid use and all-purpose ED visits and hospitalizations in community-dwelling US older adults using the 2020 National Health Interview Survey (NHIS). The findings of this study will help identify risk factors for ED visits and hospitalizations in older adults and therefore provide implications for developing intervention programs that decrease prescribed opioid use, opioid-related outcomes, ED visits, and hospitalizations in this growing population.

## Materials and methods

### The parent study design

The NHIS is administered by the National Center for Health Statistics and the CDC. As a cross-sectional household interview survey conducted yearly, the NHIS aims to monitor the health of the US population by collecting data on a broad range of health topics in non-institutionalized individuals and households with diverse demographic and socioeconomic conditions (20). The eligible participants are people residing within the 50 states and the District of Columbia at the time of the interview. Geographically clustered sampling is used to select participants thought the year. One adult per household is randomly selected to obtain in-depth information on healthcare services, health behaviors, and health status. Computer-assisted personal interviews are used for data collection. Starting in 1957, the NHIS has been the longest-ongoing national health survey in the US. The detailed methodology has been published elsewhere (21). In this study, we utilized the data of the 2020 NHIS and included older adults aged 65–85 since age of adults aged 85 and older was un-specified in the NHIS.

### Ethical considerations

This study was exempt from the University of Houston-Downtown Committee for the Protection of Human Subjects because only publicly available and de-identified data were used in this study.

### Measures

#### The prevalence of using prescribed opioids for chronic pain

The prevalence of using prescribed opioids for chronic pain was assessed by this question, “*During the past 3 months, did you take a prescription opioid to treat long-term or chronic pain, such as low back pain or neck pain, frequent headaches or migraines, or joint pain or arthritis?*” This question was only assessed among participants who had taken prescribed medications in the past 12 months and had also taken any opioids prescribed by a doctor in the past 3 months. This variable was operated as a binary variable (yes or no).

#### Independent variable: Prescribed opioid use

Participants’ prescribed opioid use was measured by this question, “*During the past 12 months, have you taken any opioid pain relievers prescribed by a doctor, dentist, or other health professional? Examples include hydrocodone, Vicodin, Norco, Lortab, oxycodone, OxyContin, Percocet, and Percodan. If you are not sure, please tell me the name of the drug and I can look it up*.” This variable was operated as a binary variable (yes or no). Participants who responded “*refused*,” “*not ascertained*,” or “*don’t know*” were excluded from the analysis.

#### Dependent variable: All-purpose (1) emergency department visits and (2) hospitalizations

Participants’ ED visits were measured by this question, “*During the past 12 months, how many times have you gone to a hospital emergency room about your health?*” This variable was operated as a categorical variable with five categories (zero, one, two, three, or four and more). Participants who responded “*refused*,” “*not ascertained*,” or “*don’t know*” were excluded from the analysis.

Participants’ hospitalization was measured by this question, “*During the past 12 months, have you been hospitalized overnight?*” This variable was operated as a binary variable (yes or no). Participants who responded “*refused*,” “*not ascertained*,” or “*don’t know*” were excluded from the analysis.

#### Covariates

To control the potential confounding factors, covariates were selected based on reviewing literature (4, 22) and included age (years), sex (male or female), ethnicity (Hispanic, African American, White, Asian, or others), education (illiterate to 12th grade with no diploma, general education development (GED) or equivalent to some college with no degree, or an associate degree and higher), employment (employed or not), general health status (good/excellent, fair, or poor), history of depression (yes or no), and living arrangement (lived alone or not). Participants who responded “*refused*,” “*not ascertained*,” or “*don’t know*” on questions related to the above covariates were excluded.

Employment was assessed by asking the participants whether they worked last week. General health status was measured by the question, “*Would you say your health in general is excellent, very good, good, fair, or poor?*” History of depression was measured by the question, “*Have you ever been told by a doctor or other health professional that you had any type of depression?*” Living arrangement was measured by asking the participants to count the number of persons 18 or older living in the household.

### Statistical analysis

The characteristics of the participants were summarized using mean (standard deviation) for continuous variables following normal distributions and median (interquartile range) for other continuous variables. Frequency (percentage) was used to summarize categorical variables. Independent *T*-tests were used to examine group differences for continuous variables. Chi-square tests were used to examine group differences for categorical variables. A linear regression model was built to examine the independent association of prescribed opioid use ED visits. Moreover, we constructed a logistic regression model to examine the independent association of prescribed opioid use with hospitalization. Both models were adjusted for age, sex, ethnicity, education, employment, general health status, history of depression, and living arrangement. Furthermore, a sensitivity test using Poisson zero-inflated model was performed to confirm the accuracy and robustness of primary analyses on ED visits. We considered a two-sided *P*-value of 0.05 or less statistically significant. All analyses were performed using R statistical software (version 2020) (23).

## Results

The sociodemographic and health information of the study participants, total and by opioid use, were shown in Table 1. The study population consisted of 8,631 older adults (mean age 74.3 [SD 6.4]). Most of them were female (58.3%), Caucasian (81.6%), did not live alone (53.9%), and unemployed/retired (82.9%). Most participants had excellent/good general health status (78.7%) with no history of depression (83.2%). In terms of prescribed opioid use, 16.3% of the participants used prescribed opioids, over half of which used prescribed opioids for chronic pain (65.1%). The participants had a mean ED visit of at least 0.34 times over the past 12 months. Additionally, 14.7% of them had hospitalizations over the past 12 months. Compared with participants not using prescribed opioids, those with prescribed opioid use were younger and more likely to be female, white, unemployed/retired and have fair or poor general health status, history of depression, hospitalizations, and ED visits.

**TABLE 1:** Participant’s sociodemographic and health information.

Characteristics	Total *n* = 8,631	Non-opioid users, *n* = 7,223 (83.7%)	Opioid users, *n* = 1,408 (16.3%)	*P*-value
Age (mean, SD)	74.3 (6.4)	74.4 (6.4)	73.6 (6.3)	**<0.001**
Female (*n*, %)	5,034(58.3)	4176 (57.8)	858 (60.6)	**0.03**
Employed/retired (*n*, %)	1,474(17.1)	1,287(17.8)	187 (13.3)	**<0.001**
Not living alone (*n*, %)	4651 (53.9)	3,885(53.8)	766 (54.4)	0.69
African American (*n*, %)	701 (8.1)	586 (8.1)	115 (8.2)	**0.01**
Asian (*n*, %)	253 (2.9)	230 (3.2)	23 (1.6)	
Hispanic (*n*, %)	527 (6.1)	453 (6.3)	74 (5.3)	
White (*n*, %)	7,045(81.6)	5,868(81.2)	1,177(86.3)	
Others (*n*, %)	105 (1.2)	86 (1.2)	19 (1.3)	
Illiterate to grade 12 (*n*, %)	841 (9.7)	706 (9.8)	135 (9.6)	**0.03**
GED to college with no degree (*n*, %)	3,672(42.5)	3,029(41.9)	643 (45.7)	
Associate degree and above (*n*, %)	4,118(47.7)	3,488(48.3)	630 (44.7)	
Excellent/good general health (*n*, %)	6,793(78.7)	5,881(81.4)	912 (64.8)	**<0.001**
Fair general health (*n*, %)	1,372(15.9)	1,046(14.5)	326 (23.2)	
Poor general health (*n*, %)	466 (5.4)	296 (4.1)	170 (12.1)	
Depression (*n*, %)	1,451(16.8)	1,091(15.1)	360 (25.6)	**<0.001**
Hospitalizations (*n*, %)	1,272(14.7)	796(11.0)	476 (38.8)	**<0.001**
Emergency visits (mean, SD)	0.34 (0.73)	0.29 (0.67)	0.58 (0.95)	**<0.001**

Bold values mean statistical significance (*P* < 0.05).

The adjusted linear regression model (Table 2) showed that prescribed opioid use, compared with no use, was independently and positively associated with increased 12-months ED visits (β = 0.22, 95% CI 0.18–0.26). Specifically, prescribed opioid use, compared with no use, increased 12-months ED visits by at least 0.22 times. In addition, advanced age, male gender, unemployment/retirement, African American (compared with being White), fair or poor general health status (compared with good/excellent general health status), and history of depression were positively associated with 12-months ED visits.

**TABLE 2:** Linear regression on the relationship between prescribed opioid use and emergency department visits.

	Coefficient	Standard error	95% CI	*T*-value	Pr (> |t|)
Age	0.01	0.001	0.007 to 0.01	7.51	**<0.001**
Male	0.03	0.02	0.002 to 0.06	2.01	**0.04**
Employed/retired	–0.05	0.02	−0.09 to 0.006	–2.26	**0.02**
Not lived alone	–0.02	0.02	−0.05 to 0.01	–1.28	0.20
**Ref: White**					
African American	0.09	0.03	0.03 to 0.14	3.10	**0.002**
Hispanic	–0.003	0.03	−0.07 to 0.06	–0.08	0.94
Asian	–0.03	0.05	−0.12 to 0.06	–0.64	0.52
Others	0.05	0.07	−0.09 to 0.18	0.71	0.48
**Ref: Associate degree and above**					
Illiterate to grade 12	0.007	0.03	−0.05 to 0.06	0.26	0.80
GED to college with no degree	0.01	0.02	−0.02 to 0.04	0.62	0.54
**Ref: Excellent/good general health status**					
Fair general health	0.23	0.02	0.19 to 0.27	10.61	**<0.001**
Poor general health	0.58	0.03	0.51 to 0.65	16.80	**<0.001**
Depression vs. no depression	0.10	0.02	0.06 to 0.14	4.91	**<0.001**
Opioid use vs. no use	0.22	0.02	0.18 to 0.26	10.59	**<0.001**

Bold values mean statistical significance (*P* < 0.05).

The adjusted logistic regression model (Table 3) revealed that prescribed opioid use, compared with no use, was independently and positively associated with increased 12-months hospitalizations (Odds Ratio [OR] 3.78, 95% CI 3.29, 4.35). In addition, advanced age, male gender, unemployment/retirement, living alone, and having fair or poor general health status (compared with good/excellent general health status) were positively associated with 12-months hospitalizations.

**TABLE 3:** Logistic regression on the relationship between prescribed opioid use and hospitalizations.

	OR	95% CI	*T*-value	Pr (> |t|)
Age	1.04	1.03−1.05	6.95	**<0.001**
Male	1.21	1.06−1.38	2.88	**0.004**
Employed/retired	0.80	0.66−0.98	–2.15	**0.03**
Not lived alone	0.83	0.73−0.94	–2.9	**0.004**
**Ref: White**				
African American	1.08	0.86−1.35	0.67	0.50
Hispanic	0.90	0.67−1.19	–0.75	0.45
Asian	0.79	0.51−1.19	–1.10	0.27
Others	0.58	0.29−1.06	–1.66	0.10
Illiterate to grade 12	0.90	0.72−1.14	0.67	0.50
GED to college with no degree	0.99	0.86−1.13	–0.15	0.88
Fair general health	2.05	1.77−2.41	9.08	**<0.001**
Poor general health	3.39	2.69−4.18	10.85	**<0.001**
Depression vs. no depression	1.02	0.87−1.21	0.27	0.78
Opioid use vs. no use	3.78	3.29−4.35	18.68	**<0.001**

Bold values mean statistical significance (*P* < 0.05).

The results of the sensitivity test, the Poisson zero-inflated model on the association between prescribed opioid use and 12-months ED visits (β = 0.22, *P* = 0.001) were consistent with that of the primary analyses. The sensitivity test result was in the Supplementary Appendix.

## Discussion

In this nationally representative sample of 8,631 community-dwelling older adults aged 65 and 85 in the US, sixteen percent of them used prescribed opioids, of which over half (65.1%) used prescribed opioids for treating chronic pain. Prescribed opioid use was independently associated with increased ED visits and hospitalizations. In addition, advanced age, male gender, unemployment/retirement, living alone, and fair or poor general health status increased older adults’ risk of ED visits and/or hospitalizations. Clinicians should identify older adults at high risk for ED visits and hospitalizations and explore non-pharmacological interventions to help them manage chronic pain. These efforts may decrease their chronic pain, opioid use, opioid use-related adverse health outcomes, ED visits, and hospitalizations.

Our study found that in older adults, prescribed opioid use was independently and positively associated with increased ED visits and hospitalization. This finding is generally consistent with those of previous studies (19, 24). A study found that the associations between prescribing hydrocodone and oxycodone and ED visits were statistically significant (both *p*-values < 0.04) (19). However, in that study, researchers did not exclusively target older adults and did not control for any covariates. In another study, researchers found that chronic opioid users had more ED visits and hospital admissions than matched non-users (24). However, that study did not target older adults and did not examine prescribed opioid use, an important source of opioid overuse in older adults. Another previous study targeting people with HIV found that people with HIV and chronic opioid therapy did not have an increased risk of ED visits or hospitalizations, compared with those with HIV but not chronic opioid therapy, after adjusting for covariates (25). However, the sample size of that study was small (*n* = 326), and the participants were only recruited from one clinic. ED visits were only collected from one medical center. In addition, people with HIV may have different demands and use for opioids compared with general older adults.

The finding of our study suggests an opioid-related impairment of life behaviors and health among older adults with unexpected and increased healthcare needs. This finding is important as there has been an increase in ED visits among older adults in the US (14). Opioid use was associated with many adverse outcomes in older adults, such as constipation, nausea, dizziness, injuries, drug-drug interactions, alcohol-drug interactions, and changes in cognition (8, 26–29). Older adults are particularly vulnerable to the effects of opioids due to their geriatric condition and possible complicated medical conditions. First, renal clearance declines by one percent every year after a person reaches 50 (30). Second, as a person ages, the metabolic activity of the liver is reduced with a decreased liver size and reduced blood flow (10). Third, as a person ages, he/she loses muscles and thus has less total body water, making opioid-related metabolites more likely to be concentrated in the blood (10, 30). Last but not least, as a person ages, his/her neurotransmitters also experience changes (30), which may decrease the therapeutic effect of opioids and increase their side effects (31). These geriatric conditions associated with opioid use may lead to increased all-purpose ED visits and hospitalizations in this population.

This study also found that more than half of the participants with prescribed opioids use used opioids for treating chronic pain (65.1%). This prevalence is consistent with previous studies, which found that chronic pain occurs in 25–85% of older adults and is the leading reason for physicians to prescribe increasing opioids for this population (32). Chronic pain indirectly increases older adults’ risk of opioid overuse and dependence. With increasing age, older adults had an increased risk of prescription opioid misuse for pain relief (33). Managing chronic pain can be very challenging and complex for this population, considering their geriatric and medical conditions (34). Especially with intensive opioid use to manage pain over decades, many older adults may have acquired opioid dependence or an opioid use disorder. Considering the negative outcomes associated with prescribed opioid use in this population, physicians should adopt a proactive, multiple-facet pain management approach with a multidisciplinary healthcare team collaboration. The approaches can include pharmacological management, physical and psychological rehabilitation, and non-pharmacological interventions to help older patients manage chronic pain (34). Regarding pharmacological management, physicians should realize that compared with younger people, older adults have a narrower therapeutic index for most pharmaceuticals with an increased likelihood of adverse drug reactions (35). Moreover, non-opioid medications such as NSAIDs, antidepressants, and anticonvulsants could be considered for managing chronic pain. NSAIDs are beneficial for treating mild to severe chronic pain, especially when used with inflammatory conditions, but they should be prescribed for the shortest duration possible in the lowest effective dose and with careful surveillance to monitor side effects (34, 36). Tricyclic antidepressants (TCAs), serotonin, and norepinephrine reuptake inhibitors (SNRIs) are antidepressants that have been approved for the treatment of chronic pain (37). However, they entail great caution in use due to potential side effects and the high prevalence of comorbidity and polypharmacy in older adults. In addition, non-pharmacological interventions, such as massage (38), music therapy (39), acupuncture (40), meditation (41), exercise (42), and yoga (43), all have been shown to provide varying degrees of relief to chronic pain. For other reasons than treating chronic pain by using prescribed opioids in our participants, we could not exactly know because of no available data. However, previous research has shown that other reasons could be to treat acute pain, cough, or diarrhea or to relax (44).

If prescribing opioids to older patients, physicians should inform older patients that (1) prescribed opioid is an important source of opioid overuse in older adults. (2) With a prolonged duration of opioid use in managing chronic pain, the analgesic efficacy of opioids is likely to decline even with an increased dose of opioids (45). (3) Prolonged opioid use leads to unwanted consequences such as tolerance, pain reinforcement, addiction, and treatment-resistant depression (46, 47). (4) One-third of older adults using opioids had opioid overuse and suffered from its associated negative outcomes (37).

Our study is subject to several limitations. First, we did not have detailed information on participants’ opioid use from family, friends, and illicit drug dealers or the exact quantity, type, and duration of their prescribed opioid use, either. Thus, the above information was not included in the analysis. However, overprescribed opioid is a significant driver of the opioid crisis (48). Based on a large national survey (336,643 participants), about half (47.4%) of US adults 65 years and older used physician prescription as the source of opioid overuse. As people age, they are less likely to use peers or family as sources of opioid overuse (49). Moreover, one-third of older adults who used opioids had opioid overuse (37). Thus, by examining older adults’ prescribed opioid use, we gained an important insight into their opioid overuse. The second limitation is that we do not know the specific reasons for their ED visits and hospitalizations. However, our findings can help target older adults at high risk of ED visits and hospitalizations and provide clinical implications for reducing older adults’ ED visits and hospitalizations. Ultimately, we would like to reduce all ED visits and hospitalizations, no matter what their causes are. The third limitation is that our study was cross-sectional. Reverse causation is possible that participants visited ED or were hospitalized first and then began to use prescribed opioids. The fourth limitation is that self-report questions were used to assess our study variables and thus were not validated questionnaires. However, previous NHIS studies involving these variables provided evidence of criterion and face validity (50).

We cannot ignore the strengths of this study. First, to our knowledge, this is the first study examining the association between prescribed opioid use and all-purpose ED visits and hospitalizations among older adults. With a large sample of nationally representative older adults in the US, the findings of this study have good generalizability. Second, the data were collected in 2020 and thus could reflect the current epidemic and problems associated with prescribed opioid use in older adults. Third, we adjusted a comprehensive list of covariates, including sociodemographic factors, mental health, and general health status, while similar previous studies did not account for all these covariates (19). Fourth, we conducted a sensitivity test to confirm the accuracy and robustness of the findings of the primary analysis. Last but not least, this study found that if a community-dwelling older adult used prescribed opioids as opposed to not using them, he/she increased the number of 12-months ED visits by at least 0.22 times. This finding could help health economics researchers and hospital managers estimate the healthcare cost associated with prescribed opioids for older adults, families, and hospitals and develop relevant policies and interventions. This finding can also alarm physicians about the potential adverse effects of opioids when they consider prescribing opioids to older patients.

Future studies are expected to assess older adults’ opioid use with more granular measures of opioids, including the type, frequency, intensity, and appropriateness of opioid use, and stratify the relationship between opioids and ED visits and hospitalizations by patients’ medical conditions.

## Conclusion

In summary, in this nationally representative sample of community-dwelling older adults, prescribed opioid use, advanced age, male gender, unemployment/retirement, African Americans, living alone, fair or poor general health status, and history of depression were positively associated with all-purpose ED visits and/or hospitalizations. Clinicians should identify older adults at high risk for ED visits and hospitalizations and encourage them to use evidence-based interventions, such as non-pharmacological pain management interventions (51), behavioral therapies (52), and counseling (53, 54) to treat chronic pain in this population. This may decrease chronic pain, opioid use, opioid-related adverse health outcomes, ED visits, and hospitalizations in this vulnerable population. This is especially important given the increasing opioid overuse and ED visits in US older adults.

## Data availability statement

The datasets presented in this study can be found in online repositories. The names of the repository/repositories and accession number(s) can be found below: The data that support the findings of this study are openly available in National Center for Health Statistics at https://www.cdc.gov/nchs/nhis/2020nhis.htm.

## Ethics statement

This study was exempt from the University of Houston-Downtown Committee Ethic approval because only public-available and de-identified data were used. Written informed consent to participate was not required.

## Author contributions

SG, CT, and ML drafted the manuscript. LW conducted the data analysis. HL provided statistical consultation. All authors read and approved the final manuscript.
