# Supplementary material for: Prescribed opioid use is associated with increased all-purpose emergency department visits and hospitalizations in community-dwelling older adults in the United States
Source: Front Psychiatry. 2022 Dec 13;13:1092199. doi: 10.3389/fpsyt.2022.1092199 (PMC9792694; doi:10.3389/fpsyt.2022.1092199)
Supplement: Supplementary file 1 [file Table_1.DOCX]

|  | | Estimate | Standard Error | Z-value | Pr (> \|Z\|) |
| --- | --- | --- | --- | --- | --- |
| Age | | 0.01 | 0.005 | 2.51 | **0.01** |
| Male | | 0.19 | 0.06 | 2.93 | **0.03** |
| Employed | | -0.15 | 0.11 | 1.39 | 0.17 |
| Not lived alone | | -0.001 | 0.06 | 0.02 | 0.98 |
| Ref: White | |  |  |  |  |
| Black | | 0.10 | 0.10 | 1.05 | 0.29 |
| Hispanic | | -0.05 | 0.13 | 0.37 | 0.71 |
| Asian | | -0.36 | 0.23 | 1.55 | 0.12 |
| Others | | -0.05 | 0.26 | 0.19 | 0.85 |
| Ref^1^: |  |  |  |  |  |
| Illiterate to grade 12 | | 0.25 | 0.10 | 2.50 | **0.01** |
| GED to college with no degree | | 0.05 | 0.07 | 0.70 | 0.49 |
| Ref: Excellent/good general health status | |  |  |  |  |
| Fair general health | | 0.51 | 0.07 | 7.08 | **<.001** |
| Poor general health | | 0.71 | 0.08 | 8.91 | **<.001** |
| Depression vs no depression  Opioid use vs no use | | 0.08  0.22 | 0.07  0.07 | 1.09  3.20 | 0.28  **0.001** |

**Appendix**

Poisson zero-inflated model on the relationship between prescribed opioid use and emergency department visits

1 means: associated degree or higher
